# Histo–Blood Group Antigen Phenotype Determines Susceptibility to Genotype-Specific Rotavirus Infections and Impacts Measures of Rotavirus Vaccine Efficacy

**DOI:** 10.1093/infdis/jiy054

**Published:** 2018-01-30

**Authors:** Benjamin Lee, Dorothy M Dickson, Allan C deCamp, E Ross Colgate, Sean A Diehl, Muhammad Ikhtear Uddin, Salma Sharmin, Shahidul Islam, Taufiqur Rahman Bhuiyan, Masud Alam, Uma Nayak, Josyf C Mychaleckyj, Mami Taniuchi, William A Petri, Rashidul Haque, Firdausi Qadri, Beth D Kirkpatrick

**Affiliations:** 1 Vaccine Testing Center, 1Department of Pediatrics; 2 Department of Medicine, University of Vermont Larner College of Medicine, Burlington; 3 Vaccine and Infectious Disease Division, Fred Hutchinson Cancer Research Center, Seattle, Washington; 4 International Centre for Diarrhoeal Disease Research, Bangladesh, Dhaka; 5 Center for Public Health Genomics and Department of Public Health Sciences; 6 Division of Infectious Diseases and International Health, University of Virginia, Charlottesville

**Keywords:** rotavirus, secretor, Lewis, vaccination, vaccine efficacy

## Abstract

**Background:**

Lewis and secretor histo–blood group antigens (HBGAs) have been associated with decreased susceptibility to P[8] genotype rotavirus (RV) infections. Efficacy of vaccines containing attenuated P[8] strains is decreased in low-income countries. Host phenotype might impact vaccine efficacy (VE) by altering susceptibility to vaccination or RV diarrhea (RVD). We performed a substudy in a monovalent RV vaccine (RV1) efficacy trial in Bangladesh to determine the impact of Lewis and secretor status on risk of RVD and VE.

**Methods:**

In infants randomized to receive RV1 or no RV1 at 10 and 17 weeks with 1 year of complete active diarrheal surveillance, we performed Lewis and secretor phenotyping and genotyped the infecting strain of each episode of RVD.

**Results:**

A vaccine containing P[8] RV protected secretors and nonsecretors similarly. However, unvaccinated nonsecretors had a reduced risk of RVD (relative risk, 0.53 [95% confidence interval, .36–.79]) mediated by complete protection from P[4] but not P[8] RVs. This effect reduced VE in nonsecretors to 31.7%, compared to 56.2% among secretors, and decreased VE for the overall cohort.

**Conclusions:**

Host HBGA status may impact VE estimates by altering susceptibility to RV in unvaccinated children; future trials should therefore account for HBGA status.

**Clinical Trials Registration:**

NCT01375647.

## Teaser

Histo–blood group antigens were associated with genotype-specific susceptibility to rotavirus infections. Nonsecretors were at decreased risk of P[4] infections, while Lewis-negative children were at increased risk of P[6] infections. These effects may impact oral rotavirus vaccine efficacy estimates.

Rotavirus (RV) remains the leading cause of infectious diarrhea among infants worldwide [1]. Oral, live-attenuated RV vaccines such as Rotarix (RV1, GlaxoSmithKline) and RotaTeq (RV5, Merck) have markedly reduced the burden of RV diarrhea (RVD), but RV still causes nearly 215000 deaths yearly among children worldwide, primarily in low-income countries (LICs) in Asia and sub-Saharan Africa [2]. For reasons not completely understood, oral RV vaccines have demonstrated reduced vaccine efficacy (VE) and effectiveness in countries with high child mortality, where disease burden remains highest [3].

RVs are triple-layered particles with an outer capsid layer comprised of VP4, a protease-sensitive protein (P) spike, and VP7, a glycoprotein (G) shell. RVs are typically classified by G and P genotypes; for example, RV1 contains a G1P[8] strain. The VP8* subunit of VP4 binds histo–blood group antigens (HBGAs) present on enterocyte surfaces, suggesting an important role for HBGAs in the pathogenesis of RV infection [4]. HGBAs are glycans ubiquitously found on mucosal surfaces and in exocrine secretions, including in the gut [5]. Increasing evidence suggests that susceptibility to infection with specific RV P genotypes is associated with HBGAs determined by secretor status and Lewis (Le) phenotype [6], controlled by the FUT2 and FUT3 genes, respectively.

An overview of secretor and Le phenotypes is provided in Supplementary Figure 1. FUT2 encodes an α[1,2]-fucosyltransferase that modifies precursor oligosaccharides to form the H-type antigen. Individuals expressing an active allele are termed secretors (*Se*), while those with a null phenotype are termed nonsecretors (*se*) and cannot express H-type antigens in the gut. FUT3 encodes an α[1,3/4]-fucosyltransferase that modifies precursor oligosaccharides or H-type antigens to form the Le^a^ or Le^b^ antigens, respectively. Lewis phenotype is thus determined by the action of both FUT2 and FUT3. However, Lewis-negative individuals (Le^–^) express neither Le^a^ nor Le^b^, irrespective of secretor status [5].

Previous studies suggest that nonsecretors and Le^–^ individuals may be resistant to infection with P[8] and P[4] RVs [7–10], whereas risk of P[6] RV infection may be increased in Le^–^ individuals [11]. This may explain the high frequency of P[6] infections in Africa, where Le^–^ phenotypes are also more frequent [11, 12]. As both RV1 and RV5 contain attenuated P[8] RVs, it has been proposed that resistance to P[8] RVs could cause resistance to vaccination and subsequent vaccine failure due to lack of protection against non-P[8] RVs. In regions with high frequencies of nonsecretors or Le^–^ individuals, this could decrease VE. Because risk among unvaccinated individuals is required to calculate VE, HBGA-mediated differences in susceptibility to RV infection among unvaccinated participants may also have unexpected implications in the analysis of RV vaccine trials.

Despite these important potential effects, the contribution of secretor status and Lewis phenotype to decreased oral RV VE in LICs has yet to be investigated. To determine the effects of secretor status and Lewis phenotype on susceptibility to natural RV infection and oral RV vaccine underperformance, we conducted a substudy among infants participating in an RV1 efficacy trial in Dhaka, Bangladesh.

## METHODS

### Study Population and Procedures

We performed a substudy within Performance of Rotavirus and Oral Polio Vaccines in Developing Countries (PROVIDE), an RV1 VE trial conducted in urban Dhaka, Bangladesh from 2010 to 2014. PROVIDE was approved by the ethical review boards of the International Centre for Diarrhoeal Disease Research, Bangladesh, the University of Vermont, and the University of Virginia and was registered at ClinicalTrials.gov (NCT01375647). All participating families provided signed informed consent. Seven hundred infants were enrolled within 7 days of life, randomized 1:1 to receive RV1 or no RV1 at 10 and 17 weeks, and followed with active community-based diarrheal surveillance. RVD was determined by RV antigen detection in diarrheal specimens using the ProSpecT enzyme immunoassay kit (Oxoid, Hampshire, UK). Severe RVD was defined as Vesikari score ≥11 [13]. Saliva was collected at 1 and 2 years of life using the SalivoBio infant swab collection kit (Salimetrics, Carlsbad, California). Study design, detailed methods, and primary efficacy results have been reported [14–16]. For this substudy, we identified infants with complete 1-year follow-up, sufficient saliva for phenotyping, and who received both doses of RV1 (for vaccinated infants) per protocol. Within this subpopulation, we performed RV genotyping and HBGA phenotyping as detailed below.

### Rotavirus P Genotyping

RVD stool specimens underwent total nucleic acid extraction using the QIAamp Fast DNA Stool Mini Kit (Qiagen, Hilden, Germany) [17]. Reverse-transcription polymerase chain reaction (RT-PCR) was performed on total nucleic acid extracts to amplify the VP8* segment of VP4 as previously described [18]. Resulting amplicons underwent Sanger sequencing using the VP4F primer on the ABI PRISM 3130xl Genetic Analyzer (Applied Biosystems, Foster City, California). Sequences were analyzed using BioEdit version 7.2.5 (Ibis BioSciences, Carlsbad, California), followed by BLAST analysis to determine the P genotype of each infecting strain.

### Secretor Status and Lewis Antigen Phenotyping

Le^a^ and Le^b^ antigen phenotyping was performed on stored saliva specimens using a dot-blot assay as previously described [19]. Infants were defined as Le^+^ if either Le^a^ or Le^b^ antigen was detected (Table 1). Secretor status was inferred from Lewis phenotyping: Le^a+b–^ infants were defined as *se*; Le^a–b+^ and Le^a+b+^ (partial-secretor) infants were defined as *Se*. Among Le^a–b–^ infants, *Ulex europaeus* agglutinin enzyme immunoassay was performed to confirm secretor status as previously described [11]. A specimen was defined as *Se* if the optical density (OD) was ≥0.09 (≥3 standard deviations above the mean OD calculated for multiple replicates of blank wells). For verification, 25 Le^a–b+^ and 27 Le^a+b–^ specimens were tested; all Le^a–b+^ were confirmed as *Se* (minimum OD = 0.114), and all Le^a+b–^ were confirmed as *se* (maximum OD = 0.071).

**Table 1.** Summary of Secretor Status and Lewis Antigen Phenotypes

Phenotype	Total (N = 550)	Unvaccinated^a^ (n = 275)	Vaccinated^a^ (n = 275)
Secretor status			
*Se*	371 (67.5)	182 (66.2)	189 (68.7)
*se*	179 (32.5)	93 (33.8)	86 (31.3)
Lewis phenotype			
Le+ (Le^a+b–^, Le^a–b+^, or Le^a+b+^)	469 (85.3)	241 (87.6)	228 (82.9)
Le^–^	81 (14.7)	34 (12.4)	47 (17.1)
Combined			
*Se*/Le^+^ (Le^a–b+^ or Le^a+b+^)	314 (57.1)	159 (57.8)	155 (56.4)
*Se*/Le^–^ (Le^a–b–^)	57 (10.4)	23 (8.4)	34 (12.4)
*se*/Le^+^ (Le^a+b–^)	155 (28.2)	82 (29.8)	73 (26.5)
*se*/Le^–^ (Le^a–b–^)	24 (4.4)	11 (4)	13 (4.7)

Data are presented as No. (%).

Abbreviations: Le^+^, Lewis-positive; Le^–^, Lewis-negative; *Se*, secretor; *se*, nonsecretor.

^a^All differences are nonsignificant.

### Statistical Analysis

Categorical outcomes were assessed using χ^2^ or Fisher exact test to estimate proportion difference with corresponding 95% confidence intervals (CIs) and associated relative risk (RR). Adjustment for multiple comparisons and corresponding calculation of adjusted *P* values (*Q* values) was performed using the Benjamini–Hochberg procedure [20]. Univariate and multivariable logistic regression was used to analyze the contributions of pertinent variables to protection from RVD and to test for interactions between variables. The primary outcome was any episode of RVD in the first year of life, except if vaccination was included as a variable, in which case the primary outcome was any episode of RVD from week 18 through week 52 of life (1 week postvaccination through 1 year). VE was calculated as [(risk among unvaccinated – risk among vaccinated) / risk among unvaccinated]. Kaplan–Meier estimators were used to calculate cumulative incidence of RVD by Lewis and secretor status. Differences between groups were tested using log-rank test. All analyses were performed using IBM SPSS software version 24 (IBM, Armonk, New York), GraphPad Prism version 7.01 (GraphPad Software, La Jolla, California), or SAS version 9.3 (SAS Institute, Cary, North Carolina). Differences were considered statistically significant at a 2-sided *P* value <.05.

## RESULTS

### Population Characteristics and P Genotypes of RVD Episodes

All 550 children identified (275 vaccinated, 275 unvaccinated) who met criteria for inclusion in this substudy were included in this analysis. Secretor status and Lewis antigen phenotypes of participants are summarized in Table 1; no differences were observed between unvaccinated and vaccinated infants. One hundred sixty-five infants experienced 174 episodes of RVD at any time during the first year of life (Table 2). One hundred eight infants experienced at least 1 episode of P[8] RVD, 19 had at least 1 episode of P[6] RVD, and 38 had at least 1 episode of P[4] RVD. Four children had 2 episodes of P[8] RVD, 4 children had P[8] RVD after infection with a different genotype, and 1 child had P[4] after an episode of P[8] RVD. Two episodes were due to P[25] RV; due to the small number of P[25] infections, these were excluded from subsequent genotype-specific analysis, but both infections occurred in Le^+^ secretors. Three untypeable infections were also excluded from genotype-specific analyses; 2 occurred in Le^+^ secretors and one in a Le^+^ nonsecretor.

**Table 2.** Infecting Rotavirus P Genotype Infants With Rotavirus Diarrhea in Year 1 of Life

Genotype	Any Rotavirus Diarrhea, Year 1 of Life					
	Unvaccinated, No. (%)			Vaccinated, No. (%)		
P genotype	First Episode (n = 103)	Second Episode (n = 5)	All Episodes (n = 108)	First Episode (n = 62)	Second Episode (n = 4)	All Episodes (n = 66)
P[4]	24 (23)	1 (20)	25 (23)	13 (21)	0 (0)	13 (20)
P[6]	9 (9)	0 (0)	9 (8)	10 (16)	0 (0)	10 (15)
P[8]	68 (66)	4 (80)	72 (67)	36 (58)	4 (100)	40 (61)
P[25]	1 (1)	0 (0)	1 (1)	1 (2)	0 (0)	1 (2)
Untypeable	1 (1)	0 (0)	1 (1)	2 (3)	0 (0)	2 (3)
	Any Rotavirus Diarrhea, Weeks 18–52 (Postvaccination)					
	Unvaccinated, No. (%)			Vaccinated, No. (%)		
P genotype	First Episode (n = 96)	Second Episode (n = 4)	Total Episodes (n = 100)	First Episode (n = 47)	Second Episode (n = 1)	Total Episodes (n = 48)
P[4]	23 (24)	1 (25)	24 (24)	11	0 (0)	11 (23)
P[6]	8 (8)	0 (0)	8 (8)	7	0 (0)	7 (15)
P[8]	65 (68)	3 (75)	68 (68)	27	1 (100)	28 (58)
P[25]	0 (0)	0 (0)	0 (0)	0 (0)	0 (0)	0 (0)
Untypeable	0 (0)	0 (0)	0 (0)	2	0 (0)	2 (4)

“First episode” refers to the first episode of rotavirus diarrhea experienced by an individual child. “Second episode” refers to the second episode of rotavirus diarrhea experienced by an individual child, and may be due to a different genotype than the first episode.

### Secretor Status and Lewis Phenotype Have Distinct Effects on Risk of Natural RV Infection Among Unvaccinated Infants

**Figure 1.**
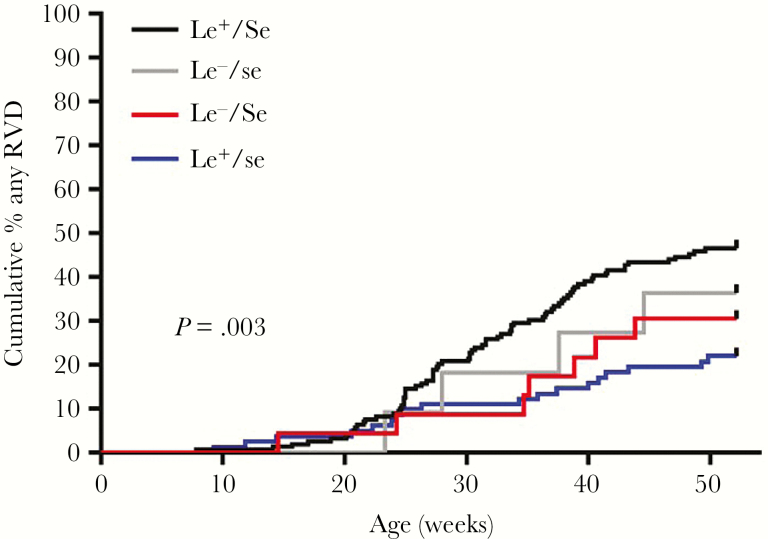
Cumulative incidence of rotavirus diarrhea (RVD) in year 1 of life among unvaccinated infants according to secretor/Lewis phenotype. The distribution pattern of RVD incidence when comparing all groups together significantly differed according to phenotype. *P* value by Mantel–Cox log-rank test. Abbreviations: Le^+^, Lewis-positive; Le^–^, Lewis-negative; RVD, rotavirus diarrhea; *Se*, secretor; *se*, nonsecretor.

We first assessed the role of secretor status and Lewis phenotype on risk of natural RV infection by analyzing the unvaccinated group. One hundred three unvaccinated infants (37.5%) had at least 1 episode of RVD; P[8] RV was most common, followed by P[4], then P[6] (Table 2). Significant differences were observed in frequency of RVD (Table 3) and time to first RVD according to combined secretor/Lewis phenotype (*P* = .003; Figure 1).

**Table 3.** Frequency of Rotavirus Diarrhea Among Unvaccinated Infants According to Secretor/Lewis Phenotype

Phenotype	Total(N = 275)	Any RVD		Severe RVD	
		(n = 103)	*Q* Value	(n = 33)	*Q* Value
*Se*/Le^+^ (Le^a–b+^ or Le^a+b+^)	159 (58)	74 (72)	0.004	24 (73)	0.041
*Se*/Le^–^ (Le^a–b–^)	23 (8)	7 (7)		1 (3)	
*se*/Le^+^ (Le^a+b–^)	82 (30)	18 (17)		5 (15)	
*se*/Le^–^ (Le^a–b–^)	11 (4)	4 (4)		3 (9)	

Data are presented as No. (%). *Q* values were calculated by adjustment of raw *P* values (Fisher exact test) for multiple comparisons by the Benjamini–Hochberg procedure.

Abbreviations: Le^+^, Lewis-positive; Le^–^, Lewis-negative; RVD, rotavirus diarrhea; *Se*, secretor; *se*, nonsecretor.

When assessed by secretor status alone, nonsecretors had a significantly reduced risk of RVD (RR, 0.53 [95% CI, .36–.79]) and were completely protected against P[4] RVD (Table 4). No differences were observed in risk of P[8] or P[6] RVD. Because a clear trend was detected for an interaction between secretor status and Lewis phenotype (*P* = .09) when tested by logistic regression, we stratified Le^+^ vs Le^–^ phenotype by secretor status (Table 4). No differences were observed in overall risk of any RVD according to Le^–^ phenotype. However, Le^–^ infants were at significantly increased risk of P[6] RVD, irrespective of secretor status (Table 4) and for severe P[6] RVD (Supplementary Table 1). Le^–^ infants also tended to have fewer episodes of any or severe P[8] RVD.

**Table 4.** Risk of Rotavirus Diarrhea According to Secretor Status, Lewis Phenotype, and Rotavirus P Genotype Among Unvaccinated Infants in the First Year of Life

Phenotype	Total	Any RVD^a^			P[8] RVD^b^			P[6] RVD^b^			P[4] RVD^b^		
	No. (%)	No. (%)	RR (95% CI)	*Q* Value	No. (%)	RR (95% CI)	*Q* Value	No. (%)	RR (95% CI)	*Q* Value	No. (%)	RR (95% CI)	*Q* Value
*Se*	182 (66)	81 (79)			51 (73)			6 (67)			25 (100)		
*se*	93 (34)	22 (21)	0.53 (.36–.79)	0.003	19 (27)	0.73 (.46–1.15)	0.22	3 (33)	0.97 (.25–3.80)	1	0 (0)	NA	<0.001
Total	275 (100)	103 (100)			70 (100)			9 (100)			25 (100)		
*Se*													
Le^+^	159 (87)	74 (91)			49 (96)			1 (17)			25 (100)		
Le^–^	23 (13)	7 (9)	0.65 (.35–1.24)	0.22	2 (4)	0.28 (.073–1.08)	0.057	5 (83)	34.3 (4.20–281)	<0.001	0 (0)	NA	0.088
Total	182 (100)	81 (100)			51 (100)			6 (100)			25 (100)		
*Se*													
Le^+^	82 (88)	18 (82)			18 (95)			0 (0)			0		
Le^–^	11 (12)	4 (18)	1.66 (.69–4.00)	0.34	1 (5)	0.41 (.061–2.81)	0.50	3 (100)	NA	0.003	0	NA	NA
Total	93 (100)	22 (100			19 (100)			3 (100)			0		

*Q* values were calculated by adjustment of raw *P* values (χ^2^ or Fisher exact test) for multiple comparisons by the Benjamini–Hochberg procedure.

Abbreviations: CI, confidence interval; Le^+^, Lewis-positive; Le^–^, Lewis-negative; NA, not applicable; RR, relative risk; RVD, rotavirus diarrhea; *Se*, secretor; *se*, nonsecretor.

^a^Refers to number of children who experienced at least 1 episode of RVD, irrespective of P genotype.

^b^Second episodes of RVD due to a different P genotype from the first are included, but second episodes due to the same P genotype are not since susceptibility to that specific P genotype had already been confirmed with the prior episode. One untypeable specimen and 1 P[25] infection were excluded. Therefore, the total number of P genotype–specific episodes differs from the total number of children with any RVD.

All 25 P[4] infections among unvaccinated infants occurred exclusively in the *Se*/Le^+^ population. To further assess whether this was mediated by secretor status or Lewis phenotype, we repeated our analyses with P[4] infections excluded. Nonsecretor status no longer conferred protection from RVD (RR, 0.80 [95% CI, .58–1.12]), but no effect was observed for Lewis phenotype (data not shown), suggesting that risk of P[4] RVD appeared to be reflected mainly by secretor status, not by Lewis phenotype.

### Secretor Status Affects RV1 Vaccine Efficacy but Lewis Phenotype Does Not

We then examined the effects of RV1 on risk of RVD according to secretor status and Lewis phenotype. One hundred forty-three infants experienced at least 1 episode of RVD from week 18 to week 52 (1 week postvaccination through 1 year of life; Table 2). Vaccination was associated with a reduced risk of any RVD (RR, 0.49 [95% CI, .36–.66]; *Q* < 0.001), RVD due to P[8] (RR, 0.42 [95% CI, .28–.63]; *Q* < 0.001), and P[4] (RR, 0.46 [95% CI, .23–.92]; *Q* = 0.031), but not P[6] (RR, 0.88 [95% CI, 0.32–2.39]; *Q* = 0.79) RV (Supplementary Table 2). Similar findings were observed for severe RVD overall and for severe P[8] RVD.

**Figure 2.**
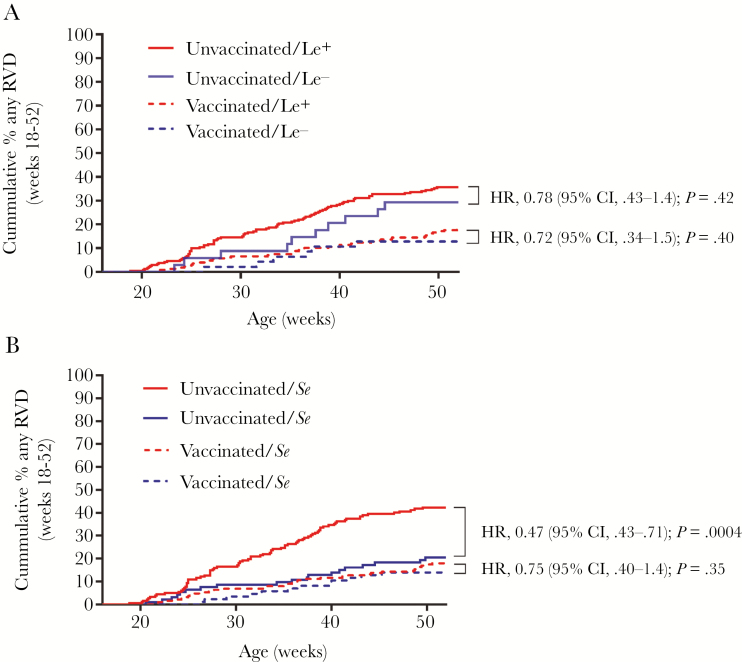
Cumulative incidence of rotavirus diarrhea (RVD) according to Lewis phenotype, secretor status, and vaccination status. Solid lines indicated unvaccinated infants; dashed lines indicate vaccinated infants. *A*, Lewis phenotype had no detectable effect modification on vaccine effect (*P* = .86), and was not associated with risk of RVD from week 18 to week 52 of life, irrespective of vaccination. *B*, Secretor status had a significant effect on RVD from week 18 to week 52 of life among unvaccinated infants but not among vaccinated infants. *P* values by Mantel–Cox log-rank test. Abbreviations: CI, confidence interval; HR, hazard ratio; Le^+^, Lewis-positive; Le^–^, Lewis-negative; RVD, rotavirus diarrhea; *Se*, secretor; *se*, nonsecretor.

In an unadjusted model, Lewis phenotype had no effect on cumulative incidence or time to first RVD for vaccinated or unvaccinated infants (Figure 2A). There was no interaction between vaccination and Lewis phenotype (*P* = .86), indicating that Lewis phenotype did not modify the vaccine effect.

In contrast, secretor status had a strong effect on RVD among unvaccinated infants (*P* = .0004) but not vaccinated infants (*P* = .35) (Figure 2B). In a multivariable logistic regression model including variables previously identified to impact risk of RVD in this [14] and similar cohorts [21] (week 18 serum zinc concentration, RV-specific immunoglobulin A [IgA] seroconversion, water treatment, exclusive breastfeeding until week 18 of life, prior RV infection, and stunting), a significant interaction was detected between vaccination and secretor status (*P* = .034). This confirmed that the effect of secretor status could not be interpreted independently of vaccination status. Therefore, separate multivariable models were run in unvaccinated and vaccinated infants to assess whether these additional variables would impact the results. After adjusting for these variables, secretor status was not associated with RVD in vaccinated infants (*P* = .5) but remained significantly associated with RVD in unvaccinated infants (*P* < .001). These results indicate that secretor status significantly modifies the effect of vaccination (ie, the effect of vaccination depended on secretor status). The risk reduction among unvaccinated nonsecretors (RR, 0.53 [95% CI, 0.36–0.80]) approached that induced by vaccination among secretors (RR, 0.44 [95% CI, .31–.62]). Although results should be interpreted with caution due to sample size, VE in nonsecretors (31.7% [95% CI, –32.2% to 64.7%]) was reduced compared to secretors (56.2% [95% CI, 38.3%–69%]). VE against severe RVD was 69.1% (95% CI, –44.7 to 93.4%]) among nonsecretors and 79.1% (95% CI, 46.1%–91.9%) among secretors.

Next, we assessed the risk of vaccine failure (ie, breakthrough RVD following vaccination) according to secretor status and Lewis phenotype; results are summarized in Table 5. There was no difference in risk of overall vaccine failure according to secretor status or Lewis phenotype. Le^–^ infants, however, had increased risk for P[6] vaccine failure, most significant among nonsecretors.

**Table 5.** Risk of Vaccine Failure According to Secretor Status, Lewis Phenotype, and Rotavirus P Genotype Among Vaccinated Infants, Weeks 18–52

	Total	Vaccine Failure^a^			P[8] Vaccine Failure^b^			P[6] Vaccine Failure^b^			P[4] Vaccine Failure^b^		
Phenotype	No. (%)	No. (%)	RR (95% CI)	*Q* Value	No. (%)	RR (95% CI)	*Q* Value	No. (%)	RR (95% CI)	*Q* Value	No. (%)	RR (95% CI)	*Q* Value
*Se*	189 (69)	35 (75)			21 (75)			2 (29)			11 (100)		
*se*	86 (31)	12 (25)	0.75 (.41–1.38)	0.49	7 (25)	0.74 (.33–1.67)	0.55	5 (71)	5.53 (1.10–27.9)	0.066	0 (0)	NA	0.066
Total	275 (100)	47 (100)			28 (100)			7 (100)			11 (100)		
*Se*													
Le^+^	155 (82)	33 (94)			21 (100)			0 (0)			11 (100)		
Le^–^	34 (18)	2 (6)	0.28 (.070–1.10)	0.066	0 (0)	NA	0.066	2 (100)	NA	0.066	0 (0)	NA	0.30
Total	189 (100)	35 (100)			21 (100)			2 (100)			11 (100)		
*se*													
Le^+^	73 (85)	8 (67)			7 (100)			1 (20)			0		
Le^–^	13 (15)	4 (33)	2.81 (.99–7.99)	0.12	0 (0)	NA	0.6	4 (80)	22.2 (2.69–183)	0.022	0	NA	NA
Total	86 (100)	12 (100)			7 (100)			5 (100)			0		

*Q* values calculated by adjustment of raw *P* values (χ^2^ or Fisher exact test) for multiple comparisons by the Benjamini–Hochberg procedure.

Abbreviations: CI, confidence interval; Le^+^, Lewis-positive; Le^–^, Lewis-negative; NA, not applicable; RR, relative risk; *Se*, secretor; *se*, nonsecretor.

^a^Children who experienced at least 1 episode of breakthrough RVD, irrespective of P genotype.

^b^Second episodes of RVD due to a different P genotype from the first are included, but second episodes due to the same P genotype are not since susceptibility to that specific P genotype had already been confirmed with the prior episode. Untypeable specimens were excluded from analysis. Therefore, the total number of P genotype–specific episodes differs from the total number of children with any RVD.

## DISCUSSION

This is the first study to investigate the effects of secretor status and Lewis phenotype on risk of RVD in South Asia and the first to assess their impact on estimates of oral RV VE. Our results provide several highly significant findings. Most importantly, we demonstrate in a cohort of Bangladeshi infants that nonsecretor status is associated with reduced risk of RVD in the absence of vaccination (Table 4; Figure 2B). This effect reduced estimates of VE, and was mediated not by reduced susceptibility to P[8] RVD as previously reported [9–11, 22–24], but rather by complete protection from P[4] RV (Table 4). These findings may have significant implications in the interpretation of past RV VE studies and in the design of future trials. In addition, we provide further evidence of increased susceptibility to P[6] RVD among Le^–^ infants (Table 4). While this did not alter overall susceptibility to RVD in this cohort, which experienced few P[6] infections, this effect could have a larger impact in regions with greater frequencies of P[6] RVD and Le^–^ individuals.

VE is calculated as [(risk among unvaccinated – risk among vaccinated) / risk among unvaccinated]. Decreased risk of infection in the unvaccinated group therefore decreases VE. In this study, resistance to RVD among unvaccinated nonsecretors decreased the risk of RVD in the unvaccinated group, thereby reducing VE. Overall per-protocol VE in PROVIDE was 51% (95% CI, 33.5%–64%) against any RVD [14]. However, we show here that VE in nonsecretors was lower (31.7%) than in secretors (56.2%). It might be expected that the effect of nonsecretor status would be equivalent across both the vaccinated and unvaccinated arms of the study and thus not have any overall effect [25]. However, the effect we observed was clearly unequal. Since RVD risk among vaccine recipients was already substantially reduced due to vaccination, the incremental effect of natural resistance was proportionally smaller, leading to a smaller risk reduction.

This unexpected mechanism by which VE calculations might be affected could have a significant impact in regions with high frequencies of nonsecretors and P[4] RV infections. This appears to be the case in Bangladesh, where we found that 32.5% of the population (Table 1) was nonsecretor, compared to 20% of the white population [26]. These results carry important implications, as they suggest that at least some degree of the decreased VE estimates observed in LICs may be due to lack of accounting for the effect of nonsecretor status on overall susceptibility to RVD in the placebo arms of VE trials. Secretor status, Lewis phenotype, and RV genotype should thus be accounted for in future oral RV vaccine trials, as has been suggested for norovirus trials [25], and previous VE estimates may need to be adjusted in regions most likely to be impacted by this effect.

The reduced risk of RVD among nonsecretors appeared to be mediated by resistance to P[4] RV. Despite being the second most common infecting strain in most regions [12], P[4] RV infections have been underrepresented in previous studies assessing RVD and secretor status or Lewis phenotype. In limited sample sizes, others have reported that P[4] RVD only occurred among secretors [8, 9, 11, 23, 24]. Our study represents the largest number of P[4] infections reported to date in this body of literature and provides evidence that nonsecretors may be naturally resistant to infection from P[4], but not P[8], RVs. Our findings are consistent with previous reports on RV genotype diversity and HBGA distributions in Bangladesh [19, 27, 28], supporting their generalizability in Bangladesh.

We found no evidence that nonsecretors were resistant to P[8] RVD (Table 4). Since RV1 contains an attenuated P[8] strain of RV, this suggests that nonsecretors are unlikely to be resistant to infection from vaccine-strain virus. This is supported by our finding that vaccinated nonsecretors were not at increased risk for vaccine failure compared to secretors and thus were afforded a similar degree of protection by RV1 (Table 5). Demonstration of similar frequencies of vaccine take by measurement of postvaccination fecal RV1 shedding and RV-specific IgA seroconversion in nonsecretors and secretors would strengthen these findings and is an important topic for future investigation. If confirmed, this would suggest that resistance to oral vaccines containing attenuated P[8] viruses is an unlikely mechanism for reduced VE in LICs. One prior study in Pakistan reported that nonsecretors had the lowest frequency of RV1 vaccine take in that population, but did not include efficacy data [29].

There are several possibilities for why we did not detect any differences in P[8] RVD according to secretor status. First, previous studies identified cases of RVD based on passive surveillance, biasing toward more severe cases. Our study identified cases via active community surveillance, potentially identifying more mild cases. It is possible that nonsecretor status may limit the severity of P[8] RVD but be permissive of milder infection. However, we did detect severe P[8] RVD in our cohort (Supplementary Table 1). It is also possible that unique strains of P[8] RV may differ in their ability to infect nonsecretors. In this cohort, 26 of 29 P[8] infections (90%) among nonsecretors were due to the same G9P[8] strain (GenBank KP902551.1). Differences in circulating P[8] RV strains might affect regional differences in susceptibility to P[8] RVs.

In contrast to secretor status, Lewis phenotype did not appear to impact VE (Table 4 and Figure 2A). However, our data further support previous findings from Burkina Faso, Nicaragua, and Tunisia that demonstrated an increased risk for P[6] RVD among Le^–^ infants [8, 11]. Similarly, we also observed that Le^–^ infants had fewer P[8] RV infections [8, 11]; this effect appeared strongest among secretors, possibly due to sample size (Table 4). However, in our cohort, this effect was offset by a markedly increased risk of P[6] RVD among Le^–^ infants (Table 4). Le^–^ infants were also protected from P[4] RVD, although this effect was weaker than that afforded by nonsecretor status. Furthermore, since removal of P[4] infections did not alter overall RVD risk according to Lewis phenotype, we submit that secretor status was the more relevant P genotype effect. However, it is possible that a Lewis phenotype effect for P[4] RVD also exists that we were unable to explicitly demonstrate.

Together, our findings underscore that the P genotype environment may have important implications for vaccine performance in different regions. In this Bangladeshi cohort, P[4] RVs had a significant impact according to secretor status. However, despite increased susceptibility among Le^–^ infants, P[6] RVs did not have a significant impact on overall risk of RVD due to the small number of P[6] RV infections observed. We also had relatively few Le^–^ infants in our study, further supporting the hypothesis that regional P[6] RV burden may reflect the population frequency of Le^–^ individuals. Furthermore, while results should be interpreted with caution due to sample size, our data suggest that RV1 conferred less protection against P[6] RVD compared to P[8] or P[4] (Table 5 and Supplementary Table 2). If this study were conducted in Africa, where P[6] infections and Le^–^ phenotypes are more common [11, 12], our results could have been changed considerably. In these locations, VE could be diminished by increased vaccine failure due to P[6] RVD. Additional efforts to characterize RV-HBGA associations in diverse settings are thus warranted [7].

This study has several limitations. First, this was a substudy of a larger VE study. Selection bias was likely limited, however, as 93% (n = 550/593) of the per-protocol parent cohort was represented. Our results were less significant for severe RVD, the primary outcome for most RV VE trials. As noted above, this is likely due to the smaller number of severe infections we encountered, limiting power. Due to inherent limitations in HBGA phenotype assays, some individuals may have been misclassified. However, our secondary validation between Lewis antigen and secretor status assays suggests this was unlikely. Confirmation via host genotype analysis would strengthen these findings but was beyond the scope of this work and is another topic for future investigation. We did not detect any mixed infections, as our sequencing approach likely selected for the most dominant strain if multiple strains were present. Although undetected mixed infections could affect these results, our results likely reflect the dominant and thus most clinically relevant genotypes. Finally, this study assessed RV1, so results may not be generalizable to other RV vaccines.

In conclusion, we demonstrate in a cohort of Bangladeshi infants that nonsecretors were at decreased risk of RVD due to complete protection from P[4] RVs. This effect could significantly impact estimates of VE, particularly in regions with high frequencies of nonsecretors and P[4] RV. We found no evidence of resistance to P[8] RVs as a mechanism for decreased VE. Le^–^ infants appeared to be protected from P[8] RVD, but this effect was offset by a markedly increased risk for RVD and vaccine failure due to P[6] infection. Secretor status, Lewis phenotype, and RV genotype should be accounted for in future oral RV vaccine trials.

## Supplementary Data

Supplementary materials are available at *The Journal of Infectious Diseases* online. Consisting of data provided by the authors to benefit the reader, the posted materials are not copyedited and are the sole responsibility of the authors, so questions or comments should be addressed to the corresponding author.

Supplementary Table 1

Supplementary Table 2

Supplementary Figure 1

## Notes

***Acknowledgments.*** We thank the PROVIDE study families and study staff for making this work possible and Timothy Hunt, Scott Tighe, and Jessica Hoffman of the University of Vermont Advanced Genome Technologies Core Facility for their assistance with RV strain sequencing.

***Potential conflicts of interest.*** All authors: No reported conflicts of interest. All authors have submitted the ICMJE Form for Disclosure of Potential Conflicts of Interest. Conflicts that the editors consider relevant to the content of the manuscript have been disclosed.

***Financial support.*** This work was supported by the Bill & Melinda Gates Foundation (grant numbers OPP1127782 to B. D. K. and OPP1017093); the National Institutes of Health (grant number AI043596 to W. A. P.); the International Centre for Diarrhoeal Disease Research, Bangladesh; and the governments of Bangladesh, Canada, Sweden, and the United Kingdom (core/unrestricted support).

Presented in part: 66th Annual Meeting of the American Society of Tropical Medicine and Hygiene, Baltimore, Maryland, 7 November 2017.
