# Erratum

**DOI:** 10.1093/infdis/jiy254

**Published:** 2018-06-04

In “Histo-Blood Group Antigen Phenotype Determines Susceptibility to Genotype-Specific Rotavirus Infections and Impacts Measures of Rotavirus Vaccine Efficacy” by Lee et al. [J Infect Dis 2018; 217:1399–407], there is an error in Figure 2A: the colors for the legend were not assigned correctly. The corrected figure is shown, below. The authors regret this error.

**Figure 2.**
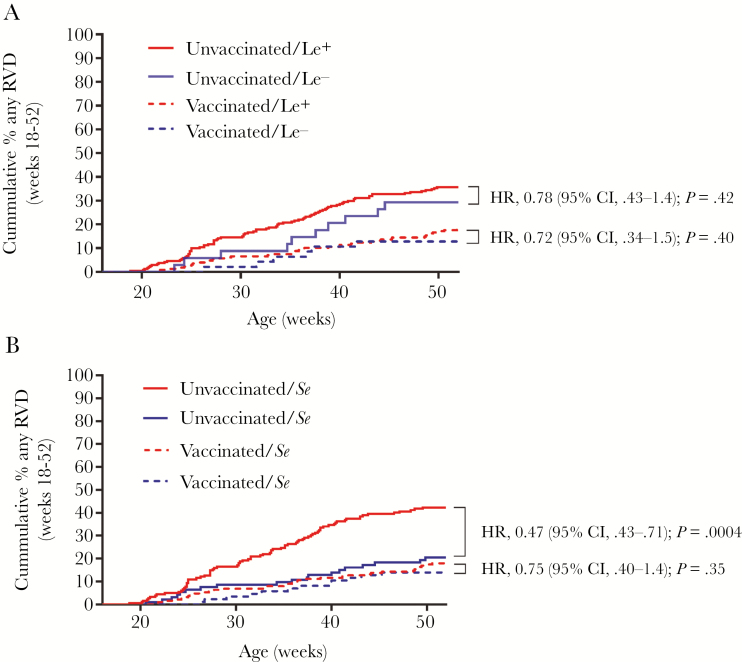
Cumulative incidence of rotavirus diarrhea (RVD) according to Lewis phenotype, secretor status, and vaccination status. Solid lines indicated unvaccinated infants; dashed lines indicate vaccinated infants. *A*, Lewis phenotype had no detectable effect modification on vaccine effect (*P* = .86), and was not associated with risk of RVD from week 18 to week 52 of life, irrespective of vaccination. *B*, Secretor status had a significant effect on RVD from week 18 to week 52 of life among unvaccinated infants but not among vaccinated infants. *P* values by Mantel–Cox log-rank test. Abbreviations: CI, confidence interval; HR, hazard ratio; Le^+^, Lewis-positive; Le^–^, Lewis-negative; RVD, rotavirus diarrhea; *Se*, secretor; *se*, nonsecretor.

