# Supplementary material for: Histo–Blood Group Antigen Phenotype Determines Susceptibility to Genotype-Specific Rotavirus Infections and Impacts Measures of Rotavirus Vaccine Efficacy
Source: J Infect Dis. 2018 Jan 30;217(9):1399–407. doi: 10.1093/infdis/jiy054 (PMC5894073; doi:10.1093/infdis/jiy054)
Supplement: Supplementary Table 1 [file jiy054_suppl_supplementary_table_1.docx]

| Supplementary Table 1. | | | Risk of Severe Rotavirus Diarrhea According to Secretor Status, Lewis Phenotype, and Rotavirus P Genotype Among Unvaccinated Infants in the First Year of Life | | | | | | | | | | | | | | |
| --- | --- | --- | --- | --- | --- | --- | --- | --- | --- | --- | --- | --- | --- | --- | --- | --- | --- |
|  | Total | Severe RVD^a^ | | | |  | Severe P[8] RVD^b^ | | |  | Severe P[6] RVD^b^ | | |  | Severe P[4] RVD^b^ | | |
|  | n (%) | n (%) | | RR (95% CI) | *Q* value |  | n (%) | RR (95% CI) | *Q* value |  | n (%) | RR (95% CI) | *Q* value |  | n (%) | RR (95% CI) | *Q* value |
| *Se* | 182 (66) | 25 (76) | |  |  |  | 15 (75) |  |  |  | 1 (25) |  |  |  | 7 (100) |  |  |
| *se* | 93 (34) | 8 (24) | | 0.63 (0.29-1.33) | 0.31 |  | 5 (25) | 0.65 (0.24-1.73) | 0.46 |  | 3 (75) | 5.84 (0.62-55) | 0.28 |  | 0 (0) | N/A | 0.28 |
| Total | 275 (100) | 33 (100) | |  |  |  | 20 (100) |  |  |  | 4 (100) |  |  |  | 7 (100) |  |  |
|  |  |  | |  |  |  |  |  |  |  |  |  |  |  |  |  |  |
| *Se* |  |  | |  |  |  |  |  |  |  |  |  |  |  |  |  |  |
| Le+ | 159 (87) | 24 (96) | |  |  |  | 15 (0) |  |  |  | 0 (0) |  |  |  | 7 (100) |  |  |
| Le─ | 23 (13) | 1 (4) | | 0.29 (0.041-2.03) | 0.31 |  | 0 (0) | N/A | 0.223 |  | 1 (100) | N/A | 0.28 |  | 0 (0) | N/A | 0.66 |
| Total | 93 (100) | 25 (100) | |  |  |  | 15 (100) |  |  |  | 1 (100) |  |  |  | 7 (100) |  |  |
|  |  |  | |  |  |  |  |  |  |  |  |  |  |  |  |  |  |
| *se* |  |  | |  |  |  |  |  |  |  |  |  |  |  |  |  |  |
| Le+ | 82 (88) | 5 (63) | |  |  |  | 5 (100) |  |  |  | 0 (0) |  |  |  | 0 |  |  |
| Le─ | 11 (12) | 3 (37) | | 4.47 (1.24-16.2) | 0.28 |  | 0 (0) | N/A | 1 |  | 3 (100) | N/A | 0.011 |  | 0 | N/A | N/A |
| Total | 93 (100) | 8 (100) | |  |  |  | 5 (100) |  |  |  |  |  |  |  | 0 |  |  |
| Abbreviations: CI, confidence interval; Le, Lewis; RR, relative risk; RVD, rotavirus diarrhea; *Se*, secretor; *se*, non-secretor. | | | | | | | | | | | | | | | | | |
| *Q* values calculated by adjustment of raw *P* values (Chi-square or Fisher’s exact test) for multiple comparisons by the Benjamini Hochberg procedure. | | | | | | | | | | | | | | | | | |
| ^a^Refers to number of children who experienced at least one episode of RVD, irrespective of P genotype. | | | | | | | | | | | | | | | | | |
| ^b^Second episodes of RVD due to a different P genotype from the first are included, but second episodes due to the same P genotype are not since susceptibility to that specific P genotype had already been confirmed with the prior episode. One untypeable specimen and one P[25] infection were excluded. Therefore, the total number of P genotype-specific episodes differs from the total number of children with any RVD. | | | | | | | | | | | | | | | | | |
