# Supplementary material for: Histo–Blood Group Antigen Phenotype Determines Susceptibility to Genotype-Specific Rotavirus Infections and Impacts Measures of Rotavirus Vaccine Efficacy
Source: J Infect Dis. 2018 Jan 30;217(9):1399–407. doi: 10.1093/infdis/jiy054 (PMC5894073; doi:10.1093/infdis/jiy054)
Supplement: Supplementary Table 2 [file jiy054_suppl_supplementary_table_2.docx]

| Supplementary Table 2. | | | Effect of RV1 Vaccination on Any and Severe Rotavirus Diarrhea, Weeks 18-52 | | | | | | | | | | | | | | |
| --- | --- | --- | --- | --- | --- | --- | --- | --- | --- | --- | --- | --- | --- | --- | --- | --- | --- |
|  | Total | Any RVD^a^ | | | |  | P[8] RVD^b^ | | |  | P[6] RVD^b^ | | |  | P[4] RVD^b^ | | |
| RV1 | n=550 (%) | n=143 (%) | | RR (95% CI) | *Q* value |  | n=95 (%) | RR (95% CI) | *Q* value |  | n=15 (%) | RR (95% CI) | *Q* value |  | n=35 (%) | RR (95% CI) | *Q* value |
| Yes | 275 (50) | 47 (33) | | 0.49 (0.36-0.66) | <0.001 |  | 28 (30) | 0.42 (0.28-0.63) | <0.001 |  | 7 (47) | 0.88 (0.32-2.39) | 0.79 |  | 11 (31) | 0.46 (0.23-0.92) | 0.031 |
| No | 275 (50) | 96 (67) | |  |  |  | 67 (70) |  |  |  | 8 (53) |  |  |  | 24 (69) |  |  |
|  |  |  | |  |  |  |  |  |  |  |  |  |  |  |  |  |  |
|  | Total | Severe RVD^a^ | | | |  | Severe P[8] RVD^b^ | | |  | Severe P[6] RVD^b^ | | |  | Severe P[4] RVD^b^ | | |
| RV1 | n=550 (%) | (n=37) (%) | | RR (95% CI) | *Q* value |  | (n=23) (%) | RR (95% CI) | *Q* value |  | (n=5) (%) | RR (95% CI) | *Q* value |  | (n=9) (%) | RR (95% CI) | *Q* value |
| Yes | 275 (50) | 7 (19) | | 0.23 (0.10-0.52) | <0.001 |  | 4 (17) | 0.21 (0.073-0.61) | 0.002 |  | 1 (20) | 0.25 (0.028-2.23) | 0.37 |  | 2 (22) | 0.29 (0.060-1.37) | 0.24 |
| No | 275 (50) | 30 (81) | |  |  |  | 19 (83) |  |  |  | 4 (80) |  |  |  | 7 (78) |  |  |
| Abbreviations: CI, confidence interval; Le, Lewis; RR, relative risk; RV1, Rotarix; RVD, rotavirus diarrhea. | | | | | | | | | | | | | | | | | |
| *Q* values calculated by adjustment of raw *P* values (Chi-square or Fisher’s exact test) for multiple comparisons by the Benjamini Hochberg procedure. | | | | | | | | | | | | | | | | | |
| ^a^Children who experienced at least one episode of breakthrough RVD, irrespective of P genotype. | | | | | | | | | | | | | | | | | |
| ^b^Second episodes of RVD due to a different P genotype from the first are included, but second episodes due to the same P genotype are not since susceptibility to that specific P genotype had already been confirmed with the prior episode. Untypeable specimens were excluded from analysis. Therefore, the total number of P genotype-specific episodes differs from the total number of children with any RVD. | | | | | | | | | | | | | | | | | |
