# Supplementary material for: Histo–Blood Group Antigen Phenotype Determines Susceptibility to Genotype-Specific Rotavirus Infections and Impacts Measures of Rotavirus Vaccine Efficacy
Source: J Infect Dis. 2018 Jan 30;217(9):1399–407. doi: 10.1093/infdis/jiy054 (PMC5894073; doi:10.1093/infdis/jiy054)
Supplement: Supplementary Figure 1 [file jiy054_suppl_supplementary_figure_1.pptx]

## Slide 1
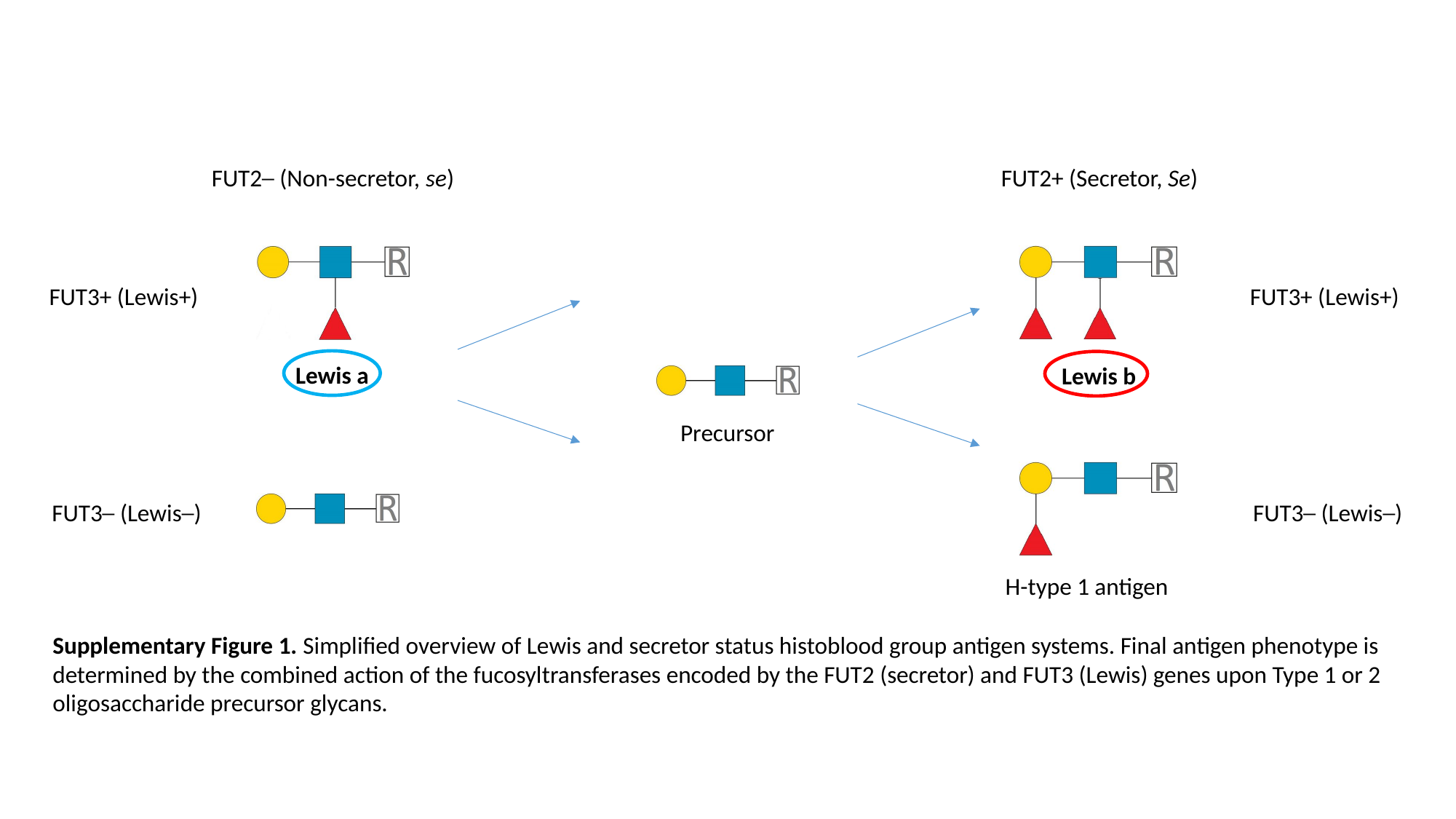

FUT2─ (Non-secretor, se)
FUT2+ (Secretor, Se)
FUT3+ (Lewis+)
FUT3+ (Lewis+)
Lewis a
Lewis b
Precursor
FUT3─ (Lewis─)
FUT3─ (Lewis─)
H-type 1 antigen
Supplementary Figure 1. Simplified overview of Lewis and secretor status histoblood group antigen systems. Final antigen phenotype is determined by the combined action of the fucosyltransferases encoded by the FUT2 (secretor) and FUT3 (Lewis) genes upon Type 1 or 2 oligosaccharide precursor glycans.
